# Occupational stress, psychological distress, physical symptoms, and their interrelationships among frontline nurses caring for COVID-19 patients in Japan

**DOI:** 10.1097/MD.0000000000031687

**Published:** 2022-12-02

**Authors:** Tomoe Nishihara, Kazufumi Yoshihara, Ayako Ohashi, Mika Kuroiwa, Nobuyuki Sudo

**Affiliations:** a Department of Psychosomatic Medicine, National Hospital Organization Fukuoka Higashi Medical Center, Fukuoka, Japan; b Department of Psychosomatic Medicine, Graduate School of Medical Sciences, Kyushu University, Fukuoka, Japan; c Department of Psychiatry, National Hospital Organization Fukuoka Higashi Medical Center, Fukuoka, Japan; d Department of Neuropsychiatry, Graduate School of Medical Sciences, Kyushu University, Fukuoka, Japan; e Department of Clinical Research Center, National Hospital Organization Fukuoka Higashi Medical Center, Fukuoka, Japan.

**Keywords:** COVID-19, frontline nurse, mental health, occupational stress, physical symptoms

## Abstract

This study aimed to identify occupational stress, psychosomatic symptoms, psychological distress, and their correlations among frontline nurses during and after the first peak of the coronavirus disease 2019 (COVID-19) outbreak in Japan. Sixteen frontline nurses, aged 25 to 52 years, working in a ward with COVID-19 patients participated in this study. Two months after the peak of the first wave of the COVID-19 outbreak in Japan, the COVID-19-related occupational stress scale (COS; questionnaire items: fear of infection and increased workload) and physical symptom scale (PS; questionnaire items: gastrointestinal symptoms, pain, appetite loss, and insomnia) were assessed. The degree of general psychological distress was evaluated using the 6-item Kessler Scale (K6). Simultaneously, participants were asked to recall their condition during the peak period of the first wave and rate it using the same scale. K6 was positively correlated with COS and PS during the peak period (rs = 0.574, *P* = .020 and rs = 0.587, *P* = .017, respectively). Increased workload was positively correlated with the K6 score both during and after the peak period (rs = 0.869, *P* < .001 and rs = 0.732, *P* = <.001, respectively) and was positively correlated with insomnia during the peak period (rs = 0.498, *P* < .05). The COS, PS, and K6 scores during the peak period were significantly higher than those after the peak period. Psychological distress at the peak was associated with PS and occupational stress. An increased workload during peak periods can cause psychological distress and insomnia. The occupational stress, PS, and psychological distress of nurses working in COVID-19 wards improved after the peak of COVID-19.

## 1. Introduction

Since the outbreak of coronavirus disease 2019 (COVID-19), the global pandemic has created tremendous challenges for healthcare workers (HCWs). To provide medical services with limited resources necessary for the ever-increasing number of cases, HCWs are forced to work long hours and handle heavy workloads. In addition, fear of infection has been reported to be a significant stressor in infectious disease epidemic.^[1–3]^ There is an urgent need to support the physical and mental health of medical personnel working in stressful conditions.

As has been widely reported in the literature, the HCWs treating COVID-19 patients have shown high levels of psychological distress, including anxiety and depression, both in other countries^[4–6]^ and in Japan.^[7]^ A report prior to the outbreak of COVID-19 indicated that physical symptoms (PS) are associated with psychological distress, such as depression and anxiety.^[8]^ During the COVID-19 outbreak, a multicenter study reported that medical staff members exhibited PS like pain or insomnia.^[9]^ In our hospital, we experienced a numerous frontline staff members who complained of anxiety and insomnia during the peak period of the first wave of the COVID outbreak. Furthermore, studies before this pandemic reported relation between insomnia and occupational stress, including increased workload, and psychological distress, such as anxiety or depression.^[10,11]^ A Japanese study also reported that depressive symptoms assessed using CES-D scores were associated with the occupational stress of nurses.^[12]^ During the peak period of the outbreak of COVID-19, in addition to a considerable increase in workload, COVID-19-related occupational stress (COS) such as increased workload and fear of infection has been reported to be associated with psychological distress.^[13]^ In our hospital, most of our frontline staff members complained about an increased workload and fear of infection with the emerging disease during the first wave of the outbreak of COVID-19. Therefore, it is important for the physical and mental health management of HCWs engaged in the treatment of COVID-19 patients to investigate their occupational stress, psychological distress, PS including insomnia, and their interrelationships.

Owing to the prolonged COVID-19 pandemic, it is increasingly important to understand the ramifications of COS and symptoms over time to provide long-term support. It is also important to identify the relations between stress and symptoms to identify targets for support at various time points as well as to clarify which symptoms should be prioritized for careful follow-up in longitudinal mental health support. To date, some studies have been conducted in Japan on the mental distress of nurses involved with COVID-19 patients.^[14,15]^ However, to our knowledge, no study has investigated in detail mental health-related symptoms, including PS and their associated factors, among Japanese frontline medical staff. This study aimed to identify the occupational stress, psychological distress, PS, and their interrelationships among Japanese frontline HCWs. In addition, we investigated how these parameters change over time.

## 2. Hypothesis

Based on previous reports, we formulated and tested the following hypotheses: there would be correlations between COS (fear of infection and increased workload), psychological distress, and PS (especially pain and insomnia) in Japanese frontline nurses during the peak period. The COS, PS, and psychological distress all would improve after the peak period.

## 3. Materials and methods

### 3.1. Study design

**Figure 1.**
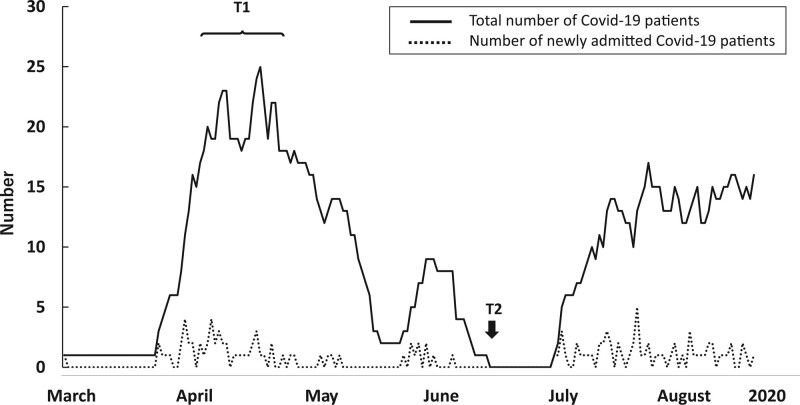
Change in the total number and number of newly admitted patients with COVID-19. The first COVID-19 epidemic peaked in April and May 2020 (T1) at the Fukuoka Higashi Medical Center National Hospital Organization. COVID-19-related occupational stress, physical symptoms, and psychological distress were assessed for frontline nurses as part of a mental support program conducted between June 23 and 28, 2020 (T2), 2 months after the peak of the first COVID-19 wave. The solid and dotted lines show the total number of COVID-19 patients hospitalized and the number of newly admitted patients, respectively. COVID-19 = coronavirus disease 2019.

This study is a cross sectional study surveying frontline nurses caring for patients with COVID-19. At our hospital, the peak of the first wave of COVID-19 admissions occurred in April and May of 2020 (T1) (Fig. 1). We assessed COS, PS, and psychological distress among frontline nurses as part of a mental support program between June 23 and 28 of 2020 (T2), 2 months after the first peak, at a time when the number of patients had been greatly reduced. The purpose of the survey was explained and all nurses agreed to participate. At that time, they were asked to recall their status during the initial peak period (T1) and record their feelings at T2.

### 3.2. Participants

Previous studies on severe acute respiratory syndrome and COVID-19 have shown that nurses are more likely to experience psychological distress than physicians because of their close contact with patients,^[5,16]^ All nurses of the special unit for the care of patients with infectious diseases at the National Hospital Organization Fukuoka Higashi Medical Center, a designated medical institution for the treatment of infectious diseases, were enrolled as participants. It is equipped with 23 negative-pressure rooms, and we started caring for COVID-19 patients in March 2020. The participants of this study were 16 frontline nurses (25–52 years old, 1 male) engaged in face-to-face care for COVID-19 patients. To determine the sample size necessary for the correlation analysis, we based our calculation on a study^[17]^ that tested the correlation between K6 and PS and found an effect size of 0.59. A sample size of 14 was calculated using these parameters (1-sided, significance level *α* = 0.05, and power = 0.80) using G*power (https://www.psychologie.hhu.de/arbeitsgruppen/allgemeine-psychologie-und-arbeitspsychologie/gpower, Heinrich-Heine-Universität Düsseldorf; North Rhine-Westphalia, Germany). Considering these missing values, the sample size for this study was set to 16. Because we could not find any previous study displaying standardized differences in the paired tests using K6 for the sample size of before-and-after comparisons, the appropriateness of the sample size was confirmed by a post hoc power analysis using the obtained results.

### 3.3. Assessment items

COS, somatic symptoms, and psychological distress were assessed using self-administered questionnaires regarding feelings over the past 30 days. Because there was no standardized questionnaire specific to healthcare professionals treating COVID-19 patients at the time, we developed 2 scales: a 2-item COS scale and a 4-item PS scale. We used 2 items of the COS scale from the questionnaire of the Japanese government’s stress check system for workers, which has proven to be predictive of long-term absence due to sickness.^[18,19]^ The validity of the 2-item COS scale and the 4-item PS scale have not been clarified, and this has been noted as a limitation. In addition, the 6-item Kessler scale (K6) was used to assess psychological distress.^[20]^ For all scales, respondents scored each item on a 5-point Likert scale ranging from 0 (seldom) to 4 (very often), as shown in Table 1.

**Table 1:** The questionnaire items used to assess occupational stress among frontline nurses working in a COVID-19 ward.

*Q. During the past 30 days*,						
	*About how often did you feel…*	*All of the time*	*Most of the time*	*Some of the time*	*A little of the time*	*None of the time*
	***COVID-19-related occupational stress* (COS**)					
	Fear of infection	4	3	2	1	0
	Increased workload	4	3	2	1	0
	***Physical symptoms* (PS**)					
	Gastrointestinal symptoms	4	3	2	1	0
	Pain (e.g., headache or shoulder stiffness)	4	3	2	1	0
	Appetite loss	4	3	2	1	0
	Insomnia	4	3	2	1	0
	***General psychological distress* (K6**)					
	Nervous	4	3	2	1	0
	Hopeless	4	3	2	1	0
	Restless or fidgety	4	3	2	1	0
	So depressed that nothing could cheer you up	4	3	2	1	0
	That everything was an effort	4	3	2	1	0
	Worthless.	4	3	2	1	0

COVID-19 = coronavirus disease 2019, K6: the 6-item Kessler Scale.

#### 3.3.1. COVID-19-related occupational stress

An increased workload was selected as COS. The labor burden was expected to increase during the peak period due to the unknown aspects of the situation and to be associated with PS and psychological distress. Another item of the COS, fear of infection, was added to assess the level of anxiety about infection, which is a common problem for healthcare professionals engaged in COVID-19 treatment. The sum of the 2 item scores was expressed as the COS score.

#### 3.3.2. Physical symptoms

Participants rated the severity of the 4 listed PS: gastrointestinal symptoms, pain, appetite loss, and insomnia. The location of pain was documented if the patient experienced pain. The sum of each item’s scores was expressed as the PS score.

#### 3.3.3. Psychological distress

To assess psychological distress, we used the K6, a self-administered questionnaire that assesses clinical depression and anxiety with established reliability and validity.^[21–24]^ It includes the following 6 items: nervousness, hopelessness, restlessness, sadness, inability to make efforts, and worthlessness. Respondents were asked to indicate their frequency over the past 30 days on a 5-point scale (each with a score of 4-0); “All of the time,” “Most of the time,” “Some of the time,” “A little of the time” and “None of the time” (total score 0–24); a score of 5 or more is considered equivalent to a psychological stress reaction, 9 or more to a mood or anxiety disorder, and 13 or more to a severe mental disorder.^[22]^

### 3.4. Statistical analysis

Statistical analysis was performed using the SPSS version 22.0 J statistical software package (IBM SPSS Statistics, Chicago, IL). Correlation analysis was performed using Spearman’s correlation analysis. The Wilcoxon signed-rank test was used to determine changes in variables from T1 to T2. post hoc power analysis was performed using the power analysis software G*Power. The criterion for statistical significance was set at *P* ≤ .05.

## 4. Results

### 4.1. Participant characteristics

All participants completed the survey. The median (lowest, 25^th^ percentile–75^th^ percentile, highest) for age, nursing experience, and duration of assignment to a ward for infectious disease patients, including before the COVID-19 epidemic, were 30 (25, 26.75–43.75, 56) years, 74 (14, 50–176, 408) months, and 23 (2, 14.75–41.00, 71) months, respectively.

### 4.2. *Correlations between occupational stress (COS), psychological distress (K6) and* PS

To examine the association between COS, K6, and PS scores, a correlation analysis between these scales was performed. At T1, positive correlations were found between the COS and K6 scores and between the K6 and PS scores (rs = 0.574, *P* = .020; rs = 0.587, *P* = .017, respectively). However, no significant correlation was found in T2 (rs = 0.424, *P* = .102; rs = 0.441, *P* = .088, respectively). The COS and PS scores showed no significant correlation at either T1 or T2 (T1 rs = 0.100, *P* = .712; T2 rs = 0.142, *P* = .600).

### 4.3. *Correlation of increased workload and fear of infection with K6 and PS scores and pain and insomnia*

The results of the correlation of the COS questionnaire items fear of infection and increased workload with K6 and PS scores and pain and insomnia, noting items in PS, are shown in Table 2. At T1, positive correlations were found between increased workload and the K6 score and between increased workload and the PS score (rs = 0.869, *P* < .001 and rs = 0.519, *P* < .05, respectively). However, in T2, a positive correlation was observed only between increased workload and the K6 score (rs = 0.732, *P* = <.001). There was no significant correlation between fear of infection and the K6 score or between fear of infection and the PS score in either T1 or T2.

**Table 2:** Correlations of COVID-19-related occupational stress (COS) (increased workload and fear of infection), general psychological distress (K6), and 2 physical symptom items (PS) (insomnia and pain) at T1 and T2.

	K6		PS		Insomnia		Pain	
	T1	T2	T1	T2	T1	T2	T1	T2
Increased workload	rs = 0.869**	rs = 0.732**	rs = 0.519*	rs = 0.219	rs = 0.498*	rs = 0.373	rs = 0.490	rs = 0.192
Fear of infection	rs = 0.205	rs = 0.036	rs = −0.209	rs = −0.069	rs = −0.238	rs = −0.024	rs = −0.166	rs = −0.161
K6	-	-	rs = 0.587*	rs = 0.441	rs = 0.656**	rs = 0.589*	rs = 0.492	rs = 0.428

The data are shown as R-values (correlation coefficient; rs) and significance levels (

* *P* < .05,

** *P* < .01).

A positive correlation was found between increased workload and insomnia scores only at T1 (rs = 0.498, *P* < .05). No significant correlation was found between fear of infection and pain or insomnia at T1 or T2. There was no correlation between pain and the K6 score at either T1 or T2; however, there was a positive correlation between insomnia and the K6 score at both T1 and T2 (rs = 0.656, *P* = .006, and rs = 0.589, *P* = .016, respectively).

### 4.4. *Changes in* COS, *psychological distress (K6) and* PS

**Figure 2.**
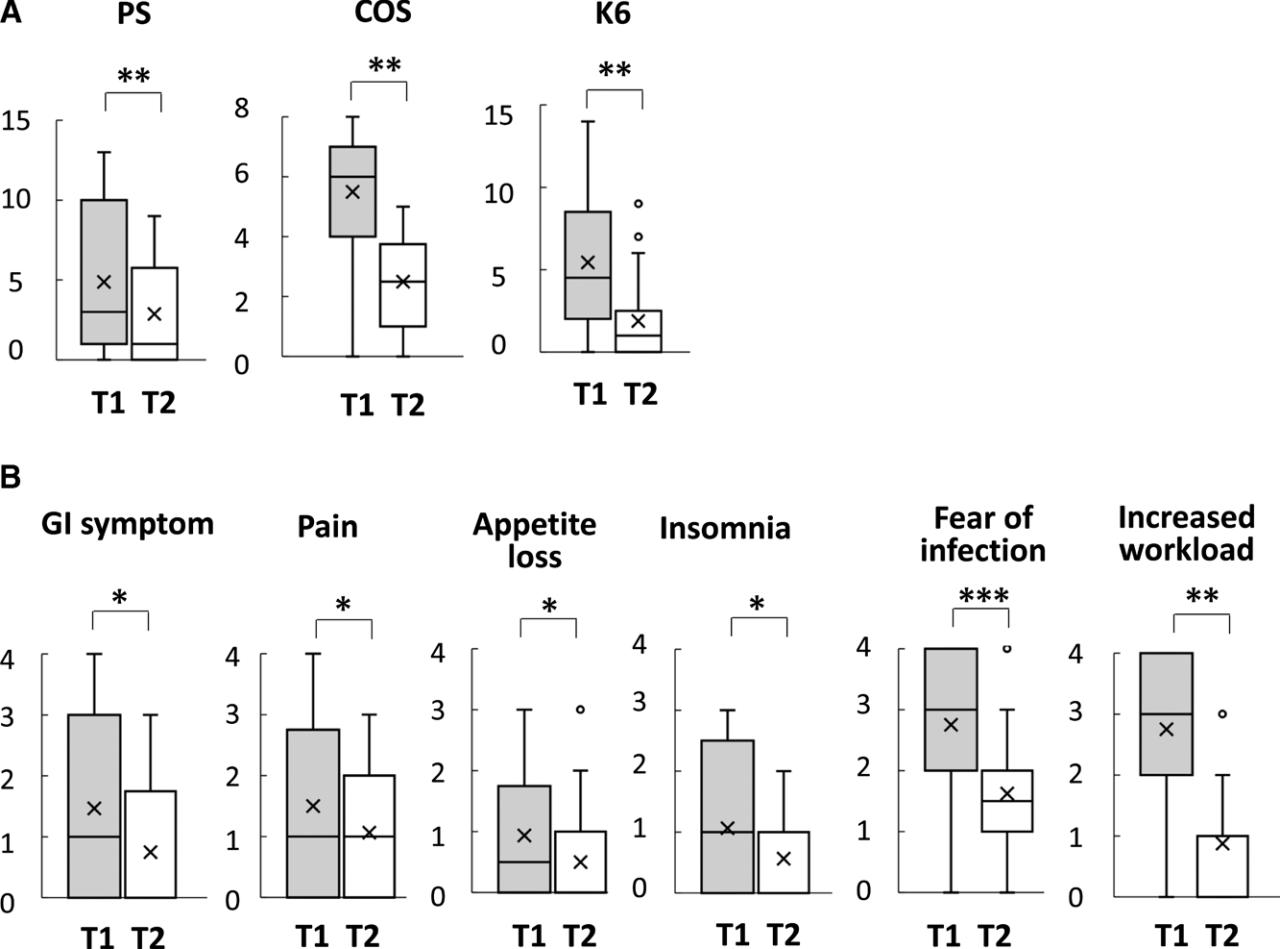
Change from T1 to T2 in COVID-19-related occupational stress, physical symptoms, and 6-item Kessler Scale. (A) The T2 scores for COVID-19-related occupational stress (COS), physical symptoms (PS), and the 6-item Kessler Scale (K6) were significantly lower than those of T1. (B) Fear of infection and increased workload of COS and gastrointestinal (GI) symptoms, pain, appetite loss, and insomnia in PS significantly decreased after the peak period. Data are shown using box-and-whisker plots. The whiskers mark the 5th and 95th percentiles, with the upper and lower ends of the boxes indicating the third and first quartile ranges of the data, respectively. The median values are expressed as the line inside the box. Asterisks indicate significant differences (*P < .05, **P < .01). COVID-19 = coronavirus disease 2019.

Changes in the scores of all questionnaires are shown in Figure 2 and Table 3. Figure 2A shows the changes in COS, PS, and K6 scores from T1 to T2. The T2 COS, PS, and K6 scores were significantly lower than those at T1(*P* < .01 for COS, *P* < .01 for PS, and *P* < .01 for K6, respectively). The COS questionnaire items fear of infection and increased workload and the PS questionnaire items gastrointestinal symptoms, pain, appetite loss, and insomnia were all significantly lower in the period after the peak (*P* < .01 for fear of infection, *P* < .01 for increased workload, *P* = .04 for gastrointestinal symptoms, *P* = .04 for pain, *P* = .04 for appetite loss, and *P* = .02 for insomnia) (Fig. 2B).

**Table 3:** Scores on each of the respective PS and COS questions and K6 scores at T1 and T2.

	T1	T2	*P*
***General psychological distress (K6***)			
Total score	4.5 (0.0, 2.0–7.5, 14.0)	1.0 (0.0, 0.0–1.5, 9.0)	<.01**
***COVID-19-related occupational stress***			
Fear of contagion	3.0 (0.0, 2.0–4.0, 4.0)	1.5 (0.0, 1.0–2.0, 4.0)	<.01**
Increase in workload	3.0 (0.0, 2.0–4.0, 4.0)	1.0 (0.0, 0.0–1.0. 3.0)	<.01**
Total score	6.0 (0.0, 4.0–7.0, 8.0)	2.5 (0.0, 1.0–3.3, 5.0)	<.01**
***Physical symptoms***			
Gastrointestinal symptom	1.0 (0.0, 0.0–2.3, 4.0)	0.0 (0.0, 0.0–1.3, 3.0)	.04*
Pain	1.0 (0.0, 0.0–2.3, 4.0)	1.0 (0.0, 0.0–2.0, 3.0)	.04*
Appetite loss	0.5 (0.0, 0.0–1.3, 3.0)	0.0 (0.0, 0.0–1.0, 3.0)	.04*
Insomnia	1.0 (0.0, 0.0–1.5, 3.0)	0.0 (0.0, 0.0–1.0, 2.0)	.02*
Total score	3.0 (0.0, 1.0–10.0, 13.0)	1.0 (0.0, 0.0–5.3, 9.0)	<.01**

Data are shown as medians (lowest, 25th percentile–75th percentile, highest) and significance levels (

* *P* < .05,

** *P* < .01).

### 4.5. *P*ost hoc *power analysis*

A post hoc power analysis was performed with the parameters (1-sided, effect size, significance level *α* = 0.05, and sample size = 16).

The power in the Spearman correlation analysis was higher than 0.80 for the significant correlations between COS and K6 score (1-β = 0.85), K6 and PS score (1-β = 0.87), increased workload and K6 score (1-β = 1.00), insomnia and K6 score (1-β = 0.95) in T1 and increased workload and K6 score (1-β = 0.99), and insomnia and K6 score in T2 (1-β = 0.87). However, the power was lower than 0.80 for a significant correlation between increased workload and PS (1-β = 0.75) at T1.

The effect size (dz) was calculated from the difference between the values of T1 and T2 and the standard deviation for the Wilcoxon single-rank sum test. The power (1-β) in the Wilcoxon single-rank sum test was higher than 0.80 for COS (dz = 1.38 and 1-β = 1.00), PS (dz = 1.43 and 1-β = 1.00), K6 (dz = 1.15 and 1-β = 1.00), fear of infection (dz = 0.98 and 1-β = 0.98), increased workload (dz = 1.72 and 1-β = 1.00) and insomnia (dz = 0.69 and 1-β = 0.82 for insomnia), but the power was lower than 0.80 for pain (dz = 0.60 and 1-β = 0.72), gastrointestinal symptoms (dz = 0.61 and 1-β = 0.73) and appetite loss (dz = 0.60 and 1-β = 0.72).

## 5. Discussion

Our survey of Japanese nurses working in inpatient wards for COVID-19 patients found the correlations between COS and psychological distress and between psychological distress and PS during the peak period, but not 2 months after the peak. Notably, increased workload and insomnia were associated with psychological distress, regardless of the peak or post-peak period, and increased workload was associated with PS during the peak period. In addition, COS, PS, and psychological distress were high during the peak period of the first wave, and then decreased after the peak had passed. The present study is notable for revealing that mental health-related PS worsened among Japanese HCWs involved in COVID-19 patients during the peak period of the outbreak and that these PS were associated with increased workload and psychological distress.

### 5.1 *Interrelation between occupational stress, psychological distress, and* PS

We revealed, for the first time, an association between occupational stress and PS among Japanese HCWs treating COVID-19 during the peak infection period. A previous report showed that occupational stressors (nursing workload and fear of infection) were associated with a high risk of anxiety and depressive symptoms.^[13]^ An association between occupational stress and psychological distress was also found in this study; however, there was no significant correlation between the fear of infection and PS. These differences may be due to differences in the subjects and/or questionnaires.

Previous studies have reported that appetite loss is correlated with depression and anxiety.^[9]^ In the current study, the total score for PS (gastrointestinal symptoms, pain, appetite loss, and insomnia) was positively correlated with the K6. This result suggests that it is necessary not only to pay attention to psychological distress but also to these PS during future waves of this or other infectious events. Although the post-peak COS, PS, and K6 scores were not correlated, the decrease in COS, PS, and K6 scores might be related to these results.

Possible interrelations were analyzed with respect to increased workload and fear of infection in COS and pain and insomnia in PS. The results showed that increased workload and insomnia were associated with psychological distress in both the peak or post-peak periods, and that increased workload was associated with insomnia during the peak period. It has been reported that a few non-working days are a risk factor for increased PHQ-9 (Patient Health Questionnaire-9; self-administered depression scale) scores among Japanese hospital workers treating COVID-19.^[25]^ Therefore, the increased workload and fewer non-work days may have exacerbated psychological distress, such as depression and PS including insomnia, in Japanese medical personnel during the peak period. Furihata reported that 41.4% of female hospital nurses in Japan had a short sleep duration of less than 6 hours.^[26]^ Therefore, along with an increased workload, insomnia should be carefully monitored, and intervention is necessary.

Pain was not associated with K6 during the peak period, which was incompatible with our hypothesis. The correlation coefficients were relatively high. It is possible that the correlation was not significant because of the small sample size. In addition, from the perspective of conceivable courses of pain, because it might take a longer time for pain to become symptomatic, future longitudinal studies are needed to clarify its association with pain.

### 5.2 *Changes in* COS, *psychological distress, and* PS

Previous studies have reported occupational stress, such as increased workload and fear of infection, and PS, such as pain and insomnia, and psychological distress among HCWs during the COVID-19 pandemic.^[4,9,27]^ These results were confirmed for the Japanese HCWs of the present study. In addition, our finding that COS, psychological distress, and PS during the peak period improved 2 months after the peak suggests that some of these mental health problems are reversible. However, if the outbreak is repeated in future waves and the period of engagement is prolonged, the results may differ. Future cross-sectional follow-up studies on the changes that occur during and after repeated waves are important.

Previous reports and our present results suggest that, to reduce psychological distress during the peak periods of COVID-19 waves, it is important to reduce COS, especially to prevent an increase in workload and not to reduce non-work days. In addition, it is important to pay careful attention to psychological distress and insomnia related to increased workloads.

The current results can be applied to nursing clinical and teaching. For example, in clinical nursing practice and education, it would be helpful for nurses to be aware that an increased workload is likely to occur when dealing with emerging infectious diseases such as COVID-19 and that both psychological distress and PS, including insomnia, will increase, which would help them with early detection and guide them in self-help for physically and mentally problems.

### 5.3 *Limitations*

Our study had several limitations. First and most importantly, the sample size is relatively small. Our results with a power lower than 0.80 may have been influenced by the small sample size. Therefore, we are currently conducting a prospective study with a larger sample size.

Secondly, the present study lacked data on baseline values, including history of PS. However, because the COS, PS, and K6 scores declined after the peak, it is thought that the baseline scores would be similar to or lower than the post-peak scores. Furthermore, we used retrospective questionnaires in part of the study, which had recall bias. However, this issue should be addressed in future studies.

We also measured the relation of anxiety to both occupational stress and psychological distress, which could affect the results. The COS item fear of infection measured specific anxiety about getting infected at work, while K6 measured nonspecific anxiety. Although anxiety was measured in both, there was no significant correlation between anxiety of infection and K6 in the current results; therefore, the possibility that this may have influenced the results appears to be small.

Additionally, we have to mention that the inferential statistical techniques used were not comprehensive. Because of the small number of participants available for the regression analysis, confounding variables could not be taken into account in the statistical analysis. Future study with a larger sample size and confounding variables will be necessary.

Finally, the participants were overwhelmingly female; however, the proportion was similar to the overall proportion of nurses in Japan (7.8% males) (Report on Public Health Administration and Services in Japan, 2018). Therefore, we consider this study to be representative of nurses working in COVID-19 wards in Japan.

## 6 Conclusion

The occupational stress, PS, and psychological distress of participating nurses working in a ward for COVID-19 patients increased during the peak period of the first wave of COVID-19 infection. An increased workload can exacerbate psychological distress and insomnia, whether or not it is during a peak period. It is important not only to pay attention to psychological distress but also to PS, including insomnia. To reduce psychological distress during peak periods of infection, it is important to control occupational stress, especially the workload and PS, including insomnia. For future clinical applications, prospective studies with larger sample sizes and control groups will be important for a high degree of validation.

## Acknowledgments

We thank the participants for their cooperation.

## Author contributions

**Conceptualization:** Tomoe Nishihara, Ayako Ohashi, Mika Kuroiwa.

**Data curation:** Tomoe Nishihara, Mika Kuroiwa.

**Formal analysis:** Tomoe Nishihara, Kazufumi Yoshihara.

**Investigation:** Tomoe Nishihara, Kazufumi Yoshihara.

**Methodology:** Kazufumi Yoshihara.

**Software:** Kazufumi Yoshihara.

**Supervision:** Kazufumi Yoshihara, Ayako Ohashi, Mika Kuroiwa, Nobuyuki Sudo.

**Writing – original draft:** Tomoe Nishihara.

**Writing – review & editing:** Kazufumi Yoshihara, Nobuyuki Sudo.
